# Genome wide association analyses to understand genetic basis of flowering and plant height under three levels of nitrogen application in *Brassica juncea* (L.) Czern & Coss

**DOI:** 10.1038/s41598-021-83689-w

**Published:** 2021-02-19

**Authors:** Javed Akhatar, Anna Goyal, Navneet Kaur, Chhaya Atri, Meenakshi Mittal, Mohini Prabha Singh, Rimaljeet Kaur, Indu Rialch, Surinder S. Banga

**Affiliations:** Department of Plant Breeding and Genetics, Punjab Agricultural University, Ludhiana, 141004 Punjab, India

**Keywords:** Plant breeding, Biotechnology, Genetics

## Abstract

Timely transition to flowering, maturity and plant height are important for agronomic adaptation and productivity of Indian mustard (*B. juncea*), which is a major edible oilseed crop of low input ecologies in Indian subcontinent. Breeding manipulation for these traits is difficult because of the involvement of multiple interacting genetic and environmental factors. Here, we report a genetic analysis of these traits using a population comprising 92 diverse genotypes of mustard. These genotypes were evaluated under deficient (N75), normal (N100) or excess (N125) conditions of nitrogen (N) application. Lower N availability induced early flowering and maturity in most genotypes, while high N conditions delayed both. A genotyping-by-sequencing approach helped to identify 406,888 SNP markers and undertake genome wide association studies (GWAS). 282 significant marker-trait associations (MTA's) were identified. We detected strong interactions between GWAS loci and nitrogen levels. Though some trait associated SNPs were detected repeatedly across fertility gradients, majority were identified under deficient or normal levels of N applications. Annotation of the genomic region (s) within ± 50 kb of the peak SNPs facilitated prediction of 30 candidate genes belonging to light perception, circadian, floral meristem identity, flowering regulation, gibberellic acid pathways and plant development. These included over one copy each of *AGL24*, *AP1*, *FVE*, *FRI*, *GID1A* and *GNC*. *FLC* and *CO* were predicted on chromosomes A02 and B08 respectively. *CDF1, CO, FLC, AGL24*, *GNC* and *FAF2* appeared to influence the variation for plant height. Our findings may help in improving phenotypic plasticity of mustard across fertility gradients through marker-assisted breeding strategies.

## Introduction

Indian mustard (*Brassica juncea*: AABB; 2n = 36) is an oilseed crop with adaptations all over the globe. It is a facultative long day (LD) plant which flowers early under long day length conditions and late during short days (SD). East European germplasms are adapted to LD conditions, while most Chinese winter type mustards are naturally biennial. Indian germplasms are winter annuals with a very short vegetative phase. However, flowering time variations exist within these geographic groups. Days to flowering can range from 20 to 145 days during Indian winters. Flowering along with the plant height are the key determinants of productivity. These are genetically complex but interrelated traits which directly or indirectly affect ecological and agronomic adaptations in plants. Both these traits are also influenced by Nitrogen (N) nutrition. Timely transition to flowering is central to the reproductive fitness as it enables reproductive phase to coincide with the conditions favourable for fruit development. Initiation of flowering requires a well-choreographed interplay of many genetic and epigenetic factors which function in concert with photoperiod, temperature, nutrient and moisture availability in the soil^1,2^. A prolonged exposure to low temperatures (vernalization) is essential for flowering in temperate plants, which respond to increasing day length conditions. In contrast, tropical plants flower at the onset of short days. This adaptive strategy prevents precocious flowering and damage to the reproductive structures. Over one hundred flowering genes are known in *Arabidopsis*^3–6^. Each of which interacts differently with the environment. *FLOWERING LOCUS T* (*FT*) and *SUPPRESSOR OF OVEREXPRESSION OF CONSTANS 1* (*SOC1*) are regulatory pivots. These genes initiate transition to flowering by increasing the expression of meristem identity genes: *APETALA 1* (*AP1*), *LEAFY* (*LFY*) and *CAULIFLOWER* (*CAL*). Upstream of *FT* and *SOC1*, in the flowering pathway, are *FLOWERING LOCUS C* (*FLC*) and *CONSTANS* (*CO*), which control floral integrators (*FT* and *FD*). Both acts differently; a stronger expression of *FLC* represses *FT* and *SOC1*. In comparison, *CO* promotes expression of *FT* and *SOC1*. Expression of floral integrators is regulated by flowering pathways, which sense environmental and developmental cues, including gibberellin levels. *FRIGIDA* (*FRI*) is a regulator of *FLC* expression^7,8^. *FLC* functions in a dosage-dependent manner and delays flowering^9^. Mutation in *FRI* reduces *FLC* expression and leads to early flowering^10^. Winter annuals possess functional copies of both *FRI* and *FLC*, whereas summer annuals show mutations in either *FRI* or *FLC* or both^11,12^. Allelic differences at these loci account for the maximum flowering time variation in *Arabidopsis*^13^. Many other genes associated with the photoperiod, vernalization, gibberellins and the autonomous pathways are also known.

Quantitative trait loci (QTLs) with large flowering effects have been mapped to different genomic regions in *B. rapa*^14^, *B. napus*^15–17^, *B. oleracea*^18^, *B. nigra*^19^ and *B. juncea*^20^. A very important flowering gene, *BrFLC2* co-localized with a flowering time QTL detected on *B. rapa* linkage group R02^21^. Forty-two small effects, but statistically significant QTLs were identified in a multi-environment study^17^. Some of these QTLs were later validated by other investigators^22,23^. However, majority of such studies used data for days to initiation of flowering as an indicator for maturity^14,20,24–26^. Genetic analysis with a doubled haploid (DH) population of *B. napus*, developed from parents differing for vernalisation responses^27^, led to the identification of flowering time QTLs in chromosomes A02, A03, A07, and C06. These included homologues of known *Arabidopsis* flowering genes: *VERNALISATION INSENSITIVE 3*, *AP1*, *FLC*, *FLT*, *CURLY LEAF*, *SHORT VEGETATIVE PHASE*, *GA3-OXIDASE* and *LEAFY*. Upadhyay et al.^28^ have reported 17 significant marker trait associations (MTA's) for six quantitative characters, including days to flowering and plant height in *B. juncea*. These explained 3.0–33.2 percent of the phenotypic variations. Availability of high-density linkage maps in *B. juncea*^29–31^ have also facilitated identification of QTLs linked to flowering time and plant height in both A- and B-genome chromosomes^32^. GWAS is an excellent alternative to biparental mapping populations as it is scalable and can detect linkage disequilibrium (LD) between molecular markers and the gene candidates. For the present studies, we used an SNP genotyped association panel of mustard to investigate genetic factors controlling variation for flowering phenology and plant height in response to external nitrogen (N) application. Phenotyping was carried out by measuring seven flowering related traits at three levels of N application and repeated over two crop seasons. Extensive variation was recorded for flowering time, maturity and plant height. Genotypes × nitrogen interactions were highly significant. Genotypes generally flowered and matured earlier at N75 as compared to N100 and N125. Genome wide association studies (GWAS) identified 282 single nucleotide polymorphism (SNP) markers that were associated with the test traits. Annotation of associated genomic regions predicted the role of several important genes related to the light perception, circadian pathway, floral meristem identity, flowering regulation, gibberellic acid pathway and general plant development. We were able to predict multiple copies of as many as six flowering genes. These outcomes emphasized the robustness of our strategy of multiple layers (N-levels in present context) of evaluating the same set of germplasm.

## Results

About 148 million clean GBS reads (56.7 Gb) were used to develop SNP genotypes for the diversity set. Various filtration steps narrowed the number to 16,250,575 SNPs with the base quality of 30. The SNPs with minor allele frequency (MAF) < 0.05 were deleted to reduce false positives. After quality control and imputation only 406,888 SNPs remained, with an overall missing rate of 20%. Final data set was then transformed to numeric values for population structure with minimal remaining missing data filled using the genotypic means of the lines. SNPs were counted at 1 Mb window size for the called genotypes along the pseudo-chromosome as displayed in Supplementary Fig. S1. A-genome harboured higher number of SNPs (212,979) as compared to B-genome (193,909). Number of SNPs per chromosome ranged from 13,267 (Chr. A10) to 40,509 (Chr. B05).

### Population structure and linkage disequilibrium

**Figure 1:**
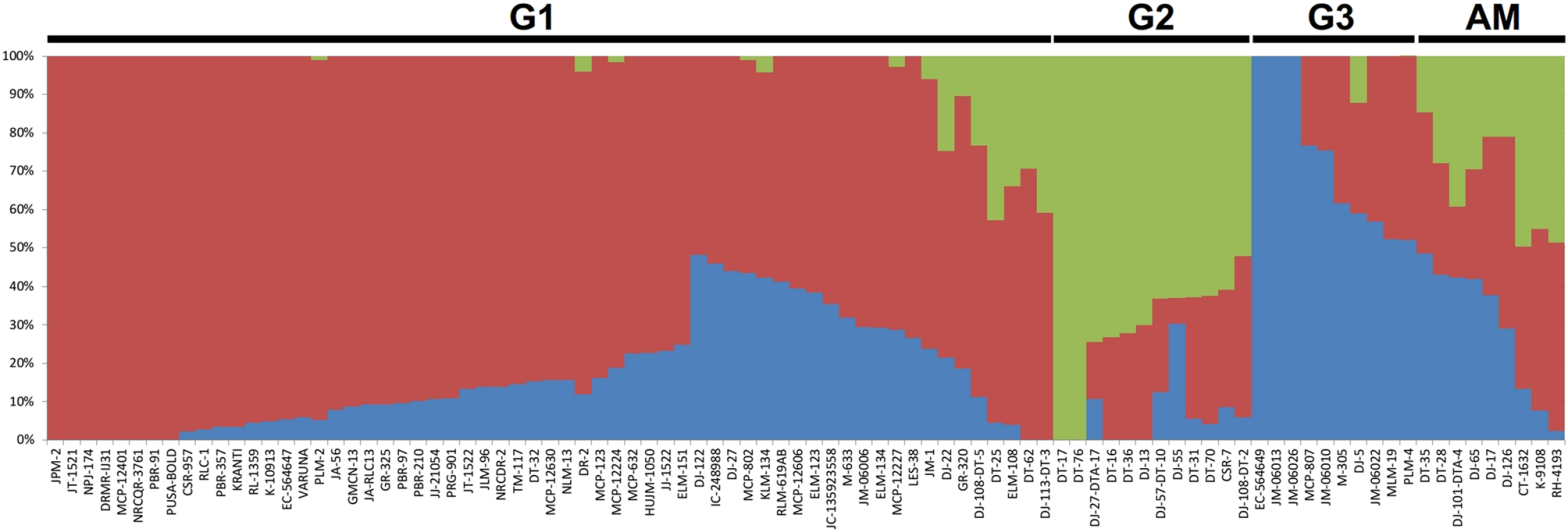
Population structure analysis suggesting three population groups in *Brassica juncea* association panel at ΔK = 3. Group 1 primarily included mustard varieties grown in India, while all resynthesized *B. juncea* genotypes with determinate inflorescence fell in the group 2. Exotic mustard genotypes formed group 3.

**Figure 2:**
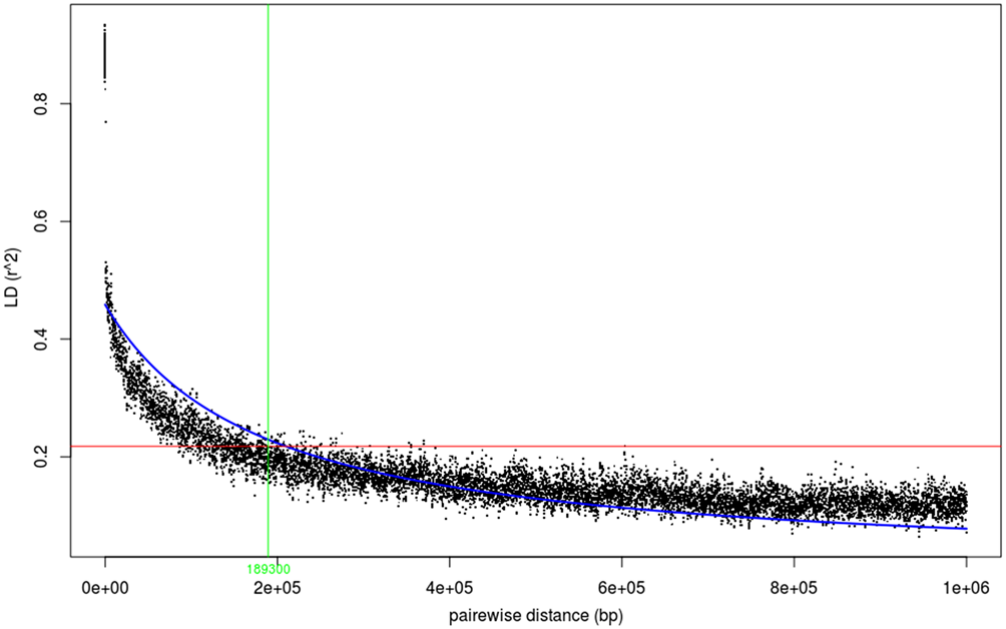
Linkage disequilibrium plotted over genetic distance**,** with *r*^2^ dropping below 0.1 at inter-marker distance beyond 1 Mb.

The genetic components of the diversity set were established by using STRUCTURE (K = 1 to 10). The value of Evanno’s ΔK peaked at K = 3. Structure (Fig. 1) included 61 lines in group one (G1), 12 in group two (G2) and 10 in group three (G3). Nine genotypes were admixtures. G1 comprised most mustard varieties grown in India with probability level higher than 0.5. All resynthesized genotypes with determinate inflorescence were included in G2. In contrast, most of exotic inbred lines fell in the group G3. Genetic distance kinship matrix provided estimates of the relatedness among individuals (K model). These allowed splitting of 92 inbred lines into three major groups (Supplementary Fig. S2). Maximum numbers of inbred lines fell in G1 (39) followed by G2 (34) and G3 (19). G1 and G2 showed relatively lower within group relatedness. Group 3 depicted maximum genetic relatedness with a range from -1.0 to -3.0 among inbred lines. Pairwise LD was estimated as *r*^*2*^ between selected set of 66,835 SNP marker genotypes in the association panel (Fig. 2). Average *r*^2^ was used as a function of inter-marker distance to estimate the LD decay in the population. Extent of LD was first evaluated for each adjacent SNP pairs. Estimates of mean *r*^2^ between 0 and 1000 kb inter-marker distance indicated that average *r*^2^ started at about 0.40 for very close markers (< 10 kb), and decayed to approximately 0.21 for SNPs as distant as 200 Kb. The mean *r*^2^ dropped below 0.1 when inter-marker distance increased beyond 1 Mb.

### Analysis of the phenotypic data

**Figure 3:**
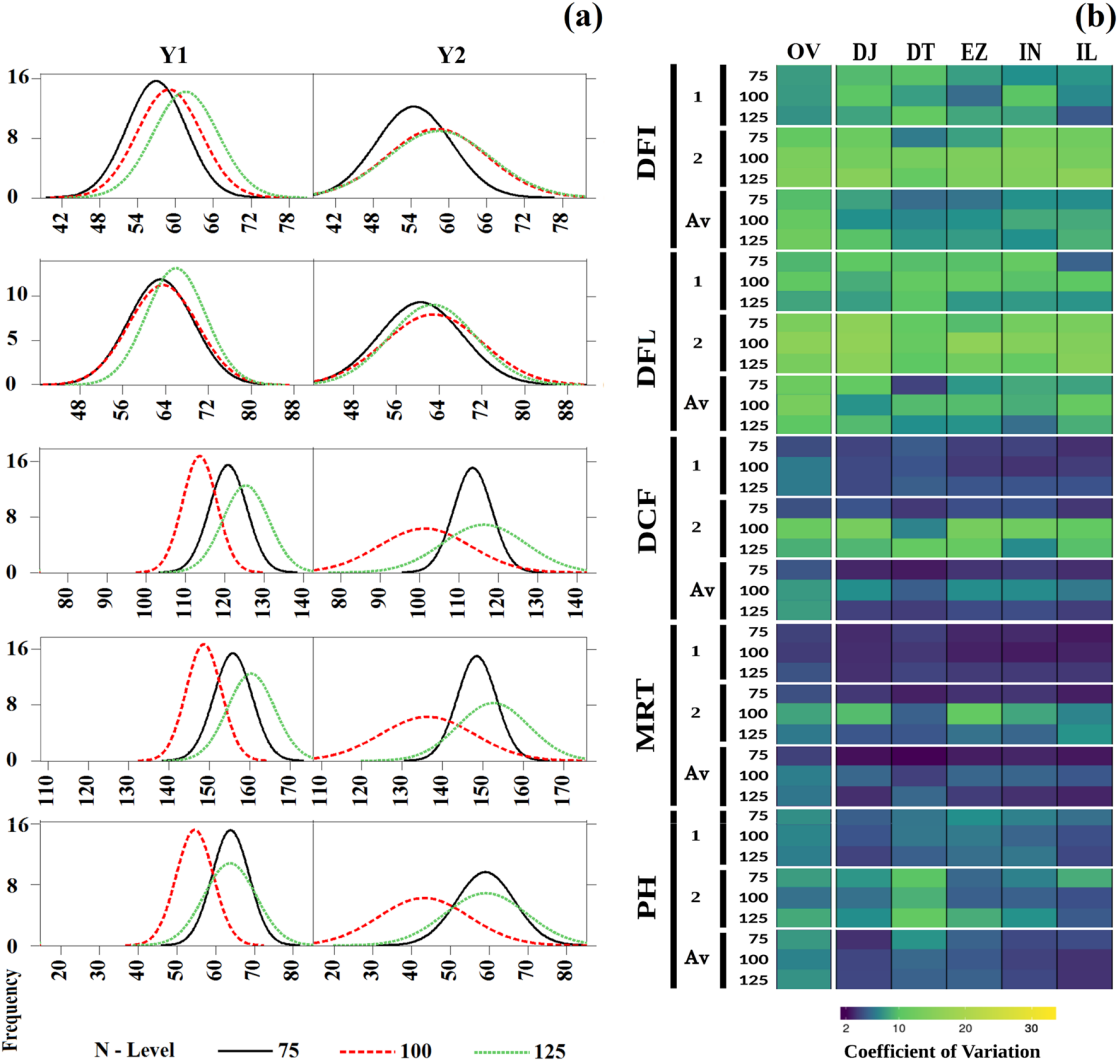
**(a) **Phenotypic variations for days to flowering initiation (DFI), days to 50% flowering (DFL), days to complete flowering (DCF), days to maturity (MRT) and plant height (PH) in *Brassica juncea* association panel; **(b)** coefficient of variations for flowering traits as compared to the days to maturity and plant height.

**Figure 4:**
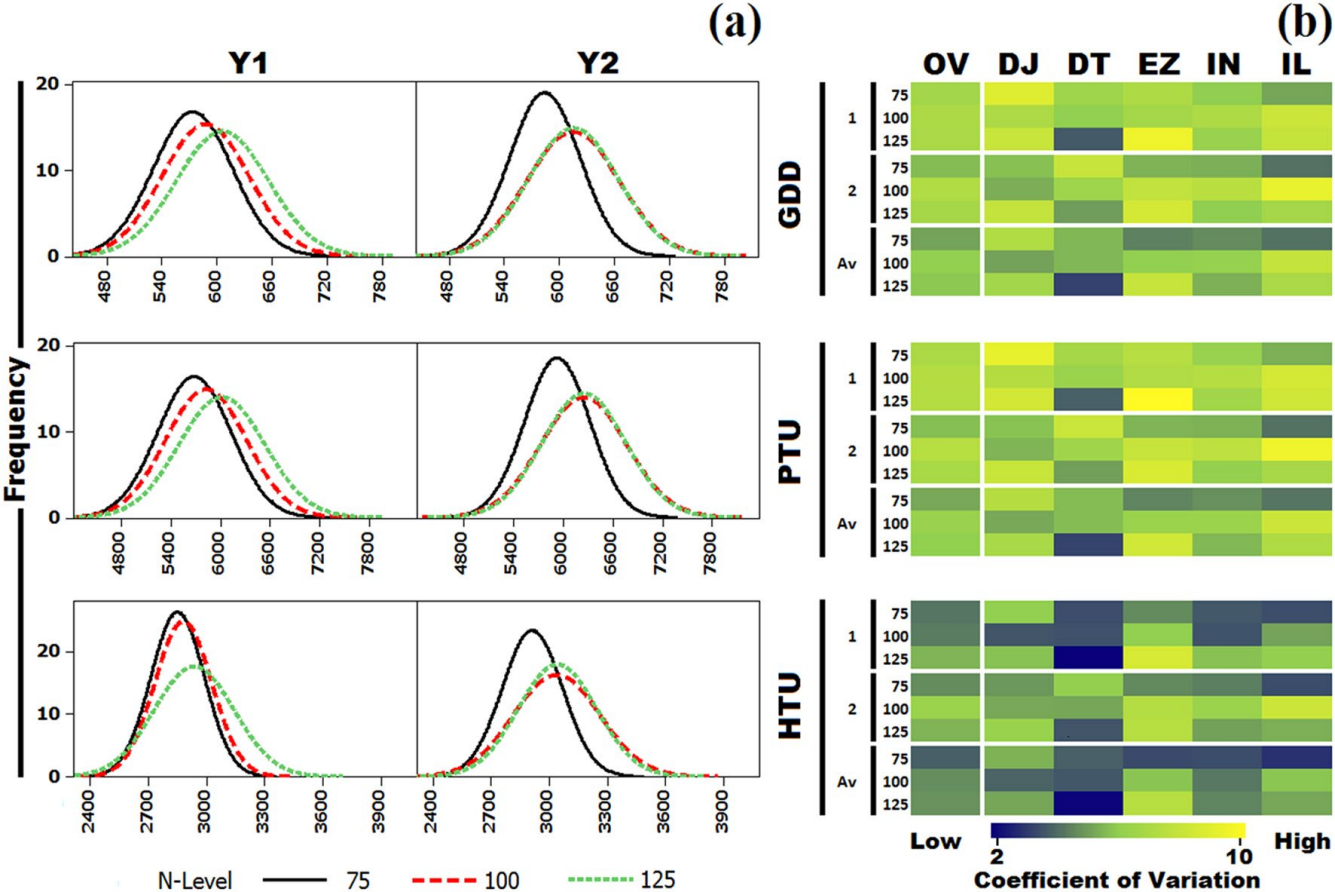
**(a)** Phenotypic variations for growing degree days (GDD), photo-thermal units (PTU) and Helios thermal unit (HTU) at initiation of flowering in the *B. juncea* diversity panel; **(b)** coefficient of variations for GDD, PTU and HTU.

High phenotypic variations were observed for flowering time {DFI (days to flower initiation), DFL (days to fifty percent flowering), DCF (days to complete flowering)}, MRT (days to maturity), GDD (growing degree days), HTU (helio-thermal unit), PTU (photo-thermal units) and PH (plant height) at three levels of N application (Fig. 3a). Analysis of variance for the design indicated strong genotypic and genotype × environment (N-levels and years) interactions (Supplementary Table S1). Distribution of the phenotypic variations was largely normal during year 1 (Y1) and year 2 (Y2). However, the distribution of variation was somewhat skewed during Y2 for DCF and PH. Trait coefficients of variation in the diversity panel and across different sub-groups within diversity panel are presented in Fig. 3b. Flowering traits had higher coefficient of variation as compared to maturity and plant height. Impact of N-level and crop season on coefficient of variation was also apparent. DFI ranged from 44.54 to 73.31 days across nitrogen levels during Y1 and 41.00 to 81.00 days during Y2. Germplasm lines generally flowered earlier at N75 as compared to N100 and N125. RLM-619-AB, M-305, DJ-108 DT-2 were consistently early flowering genotypes. DFL ranged from 49.29 to 79.80 days during the Y1 as compared to 45.50 to 89.00 days during the Y2 (Table 1, Fig. 3a). DCF varied from 103.00 to 138.19 days and 75.50 to 132.50 during the Y1 and Y2 respectively. MRT ranged between 138.00 to 173.19 days (Av. 154.92 days) during Y1. This was in contrast to 110.50–167.50 days (Av. 145.72 days) during the Y2. DJ-55, MLM-19, CSR-957 and EC-56–4647 were earliest to mature. GDD ranged from 446.85 to 792.00 degree days during the Y1 and from 456.95 to 811.50 degree days during the Y2 (Fig. 4a). PTU varied from 4392.10 to 7970.90 units and 4640.15 to 8303.00 units during the Y1 and Y2, respectively. HTU averaged at 2887.08 units (2367.25 to 3934.50) for the Y1 and 2997.50 units (2360.60 to 4013.00) units for Y2. Coefficients of variations were higher for GDD and PTU as compared to HTU (Fig. 4b). PH varied between 173.28 and 254.37 cm during the Y1 with mean value of 217.92 cm, which was almost at par with the values recorded during the Y2 (Fig. 3a). As is expected for functionally and developmentally related traits, strong phenotypic correlations were observed among the traits investigated. DFI, DFL, GDD, HTU, PTU and PH were strongly correlated in across years and N-levels (Supplementary Fig. S3).

**Table 1:** Basic descriptive statistics of flowering traits in *B. juncea* diversity fix foundation set (*BjDFFS*) during 2015–16 and 2016–17.

Traits	Year	Mean ± standard error			Range						C.V			Significance		
		N75	N100	N125	N75		N100		N125		N75	N100	N125	N75	N100	N125
					Min	Max	Min	Max	Min	Max						
Days to flower initiation (DFI)	1	56.97 ± 0.49	59.00 ± 0.53	61.62 ± 0.54	44.54	69.39	48.35	69.92	50.42	73.31	8.24	8.57	8.38	**	**	**
	2	54.39 ± 0.63	58.05 ± 0.83	58.38 ± 0.85	41.50	68.50	41.00	77.50	43.00	81.00	11.06	13.72	14.05	-	-	-
	P	55.68 ± 0.41	58.48 ± 0.50	60.00 ± 0.51	46.78	64.37	47.43	69.52	49.43	71.09	7.00	8.17	8.18	**	**	**
Days to 50% flowering (DFL)	1	63.13 ± 0.64	63.56 ± 0.67	65.94 ± 0.58	49.29	78.83	51.90	78.00	53.11	79.80	9.72	10.19	8.40	**	**	**
	2	60.56 ± 0.82	62.87 ± 0.96	62.72 ± 0.84	46.00	79.50	45.50	89.00	47.00	81.00	12.92	14.59	12.84	-	-	-
	P	61.85 ± 0.53	63.14 ± 0.62	64.36 ± 0.51	51.65	74.07	50.72	75.65	53.84	76.06	8.27	9.36	7.59	**	**	**
Days to complete flowering (DCF)	1	120.84 ± 0.49	113.60 ± 0.45	125.33 ± 0.61	110.12	131.53	103.00	122.00	113.41	138.19	3.92	3.84	4.66	**	**	**
	2	116.31 ± 1.11	101.19 ± 1.21	162.02 ± 0.10	103.50	125.25	75.50	123.50	81.00	132.50	4.29	11.44	9.15	-	-	-
	P	115.75 ± 0.41	109.89 ± 0.77	108.96 ± 0.47	106.63	123.00	92.37	125.22	98.98	120.23	3.36	6.79	4.18	-	-	-
Days to maturity (MRT)	1	155.84 ± 0.49	148.60 ± 0.46	160.33 ± 0.61	145.12	166.53	138.0	157.00	148.41	173.19	3.04	2.94	3.64	**	-	**
	2	148.44 ± 0.51	136.19 ± 1.21	152.53 ± 0.92	138.50	160.25	110.50	158.50	116.00	167.50	3.28	8.50	5.78	-	-	**
	P	150.75 ± 0.46	144.89 ± 0.78	144.70 ± 0.56	141.63	158.00	127.37	160.22	133.98	169.00	2.58	5.15	3.17	-	-	-
Growing degree days (GDD)	1	573.75 ± 16.10	587.62 ± 16.15	606.19 ± 20.65	446.85	677.80	476.50	751.45	484.90	792.00	3.97	3.89	4.82	**	**	**
	2	584.25 ± 17.24	614.20 ± 31.58	615.95 ± 24.31	456.95	691.30	480.75	777.85	501.70	811.50	4.17	7.27	5.58	**	**	**
	P	579.00 ± 20.60	587.62 ± 16.15	611.07 ± 22.66	512.40	653.40	476.50	751.45	528.50	738.75	7.12	3.89	7.42	**	**	**
Photo-thermal unit (HTU)	1	5681.68 ± 166.37	5823.59 ± 165.71	6015.06 ± 212.67	4392.10	6758.25	4691.15	7533.50	4775.85	7970.90	4.14	4.02	5.00	**	**	**
	2	5930.92 ± 177.26	6239.79 ± 329.61	6257.40 ± 250.94	4640.15	7032.45	4880.30	7948.05	5091.80	8303.00	4.23	7.47	5.67	**	**	**
	P	5806.30 ± 211.21	5823.59 ± 165.71	6136.23 ± 234.17	5055.10	6642.63	4691.15	7533.50	5218.52	7525.60	7.28	4.02	7.63	**	**	**
Helio-thermal unit (PTU)	1	2848.08 ± 89.86	2879.31 ± 56.88	2933.86 ± 78.68	2367.25	3245.70	2572.15	3690.80	2598.70	3934.50	4.46	2.79	3.79	**	**	**
	2	2914.45 ± 69.38	3033.91 ± 154.71	3044.14 ± 105.61	2360.60	3349.20	2483.05	3810.60	2640.90	4013.00	3.37	7.21	4.91	**	**	**
	P	2881.27 ± 78.68	2879.31 ± 56.88	2989.00 ± 94.93	2612.77	3198.58	2572.15	3690.80	2731.50	3557.18	5.46	2.79	6.35	**	**	**
Plant height (PH)	1	210.29 ± 1.39	216.37 ± 1.23	227.11 ± 1.27	173.28	249.87	187.17	243.15	201.02	254.37	6.36	5.47	5.38	**	**	**
	2	207.57 ± 1.69	206.51 ± 1.37	215.93 ± 1.83	176.40	244.00	165.15	235.80	161.90	260.90	7.82	6.35	8.12	-	*	-
	P	208.81 ± 1.10	211.37 ± 1.02	221.58 ± 1.04	181.48	232.91	182.52	235.93	198.26	247.41	5.06	4.61	4.50	**	-	**

*P* pooled.

**Significant at 1% level.

*Significant at 5% probability level. Analysis of variance for the design was used for the test of significance.

### GWAS studies

GWAS was conducted to identify marker trait associations (MTA's) based on the multi-environment phenotyping data (Table 2) using GAPIT3. An ideal model is expected to show a fair degree of uniformity between the observed and expected p-values in the plot. We compared p values [observed − log10 (p-value)] and their expected ranked values [expected − log10 (p-value)] through quantile–quantile (QQ) plots to test the predictability of applied GWAS models, over all environments (Supplementary Fig. S4). An arbitrary threshold value of –log 10 (p-value ≥ 3.0) allowed detection of 282 MTA's (Supplementary Fig. S5). Genomic regions around the identified SNPs were further annotated to decipher trait related genes. Thirty-eight MTA's led to prediction of genes involved in light perception pathway. Phenotypic variation explained ranged from 10.69 to 20.23%. Of these, 11 MTA's were detected for chromosome A02 in the genomic region 36,472,064–36,487,130 bp. These MTA's explained variation for GDD, PTU and PH at N75 and N100 levels of nitrogen application. Annotation predicted *CYCLING DOF FACTOR* 1 (*CDF1)-AT5G62430* in the vicinity. 10 MTA's involving chromosome A08 were found associated with *FD-AT4G35900* at N100 for DFL. *CONSTANS (CO)-AT5G15840* was envisaged on chromosome B08. *CRY1 (CRYPTOCHROME 1)-AT4G08920* was predicted through 15 MTA's, identified in the region 338,948–346,586 on B03. These MTA's involved two flowering traits (DFI, DFL) and were common across three levels of N application. Two Circadian pathway genes, *CKB4-AT2G44680* and *AT1G22770-GI (GIGANTEA)* were envisioned for DFL at N75 for chromosomes A05 and A09, respectively. These associations explained 9.79–11.79% of phenotypic variation for flowering traits.

**Table 2:** Genome wide association studies to predict candidate genes associated with flowering, maturity and plant height.

Pathway	Predicted gene	Associated traits	N-level	Chr	SNP position	NSNPs	Dis. (kb)	R^2^ (%)	Gene function
Light perception	*CDF1-AT5G62430*	GDD, PTU	N75, N100	A02	36,472,064–36,472,185	6	14.33	14.31	Regulates light perception and signalling
		PH	N75		36,486,270–36,487,130	5	0.03	19.18	
	*FD-AT4G35900*	DFL	N100	A08	18,952,302–18,955,620	10	38.68	10.69	Regulates light perception and signalling and flowering transition
	*CO-AT5G15840*	PH	N125	B08	9,748,710–9,748,737	2	43.93	13.95	Regulates light perception, signalling and flowering time
	*CRY1-AT4G08920*	DFI, DFL	N75, N100, N125	B03	338,948–346,586	15	2.01	20.23	Regulates light perception and signalling
Circadian pathway	*CKB4-AT2G44680*	DFL	N75	A05	2,937,409–2,945,567	5	16.89	9.79	Regulation of circadian clock
	*GI-AT1G22770*	DFL	N75	A09	39,780,608–39,780,959	3	56.1	11.79	Regulation of circadian rhythm and photoperiodic flowering
Floral meristem identity genes	*AGL24-AT4G24540*	GDD	N100	A02	34,934,199–34,934,540	6	26.26	12.76	Regulates Flower development and meristem identity and mediates floral transition in response to vernalization
		GDD, HTU, PTU	N125	A03	33,169,034–33,177,336	4	12.43	3.65	
		DCF, MRT	N125	B02	59,088,464	1	24.51	10.57	
		PH	N75		64,496,077–64,496,528	3	3.5	19.27	
		DFI, DFL	N100		64,497,529–64,499,514	3	0.21	13.19	
	*AP1-AT1G69120*	PTU	N75	A02	34,755,893–34,758,537	5	0.04	11.48	Regulates flower development and meristem identity
		HTU	N100	B02	59,351,856–59,353,415	8	25.11	12.22	
	*AP2-AT4G36920*	DFL	N75		66,542,016–66,546,535	6	28.89	9.8	Promotes early floral meristem identity
	*JMJ14-AT4G20400*	DFL	N100	B03	10,133,662–10,134,104	2	6.84	12.33	Inhibit the floral transition
Flowering regulation	*KHZ1-KHZ2-AT5G06770*	GDD, HTU, PTU	N125	A02	1,202,251–1,217,865	78	2.56	3.27	Regulates floral transition redundantly
	*FLOR1-AT3G12145*	GDD, HTU, PTU	N125		1,202,251–1,217,865	78	2.96	3.27	
	*FLC-AT5G10140*	PH	N100		1,762,477	1	20.82	10.61	Represses floral transition
	*FLM-AT1G77080*	DFL	N125		37,090,239–37,093,082	23	38.02	15.59	Prevents premature flowering
		GDD, HTU, PTU	N100		37,133,361–37,136,964	5	0.82	14.10	
	*FLD-AT3G10390*	GDD,HTU	N75	A03	19,913,046–19,923,910	8	0.94	3.12	Up-regulation of *FLC* expression
	*AGL6-AT2G45650*	DCF, MRT	N75	A05	3,354,215	1	13.69	20.63	Regulates flowering time
	*SOC1-AT2G45660*	DCF, MRT	N75		3,354,890–3,357,582	3	17.80	18.57	
	*COP1-AT2G32950*	DCF, MRT	N75		7,544,327–7,546,996	24	1.91	18.94	Repressor of photomorphogenesis
	*FKF1-AT1G68050*	DFI	N100	A07	1,786,248	1	15.08	13.82	Regulates transition to flowering
	*FT-AT1G65480*	MRT			33,488,659	1	21.21	12.46	Promotes the transition from vegetative growth to flowering
		DFL		B06	6,716,803–6,718,200	5	33.81	12.23	
	*ARP6-AT3G33520*	DCF	N75, N100	A08	52,619–52,842	10	25.97	17.18	Regulates flowering and *FLC* gene
	*FVE-AT2G19520*	PH	N125	A09	10,079,474	1	3.43	15.75	Regulates flowering
			N100	B04	16,336,556–16,341,785	7	0.66	10.19	
	*FRI-AT4G00650*	MRT	N125	A10	6,054,217–6,054,243	2	39.16	15.52	
		DCF, PH	N75	B08	16,066,635	1	30.42	16.04	
	*MAF5-AT5G65080*	DCF	N125	B06	8,515,215–8,524,113	6	46.85	12.47	Prevents premature flowering
	*HAM1-AT5G64610*	DCF, MRT	N75	B07	11,563,802–11,564,171	5	0.61	16.43	Essential for gametophyte development
Gibberellin pathway	*GID1A-AT3G05120*	DFI	N75	A05	30,987,755–30,990,279	3	0.42	16.51	Functions as soluble gibberellin receptor
		DFL, DCF	N75, N125	B02	62,297,219–62,297,220	2	35.29	10.61	
	*GA1-AT4G02780*	DCF, MRT	N100	B02	46,682,290	1	43.96	13.04	Gibberellin biosynthesis
		DFI	N100	B08	14,118,234	1	5.71	11.71	
	*FAF2-AT1G03170*	DFI	N125	B03	115,277	1	0.06	17.53	Regulates shoot meristem size
	*ATJ3-AT3G44110*	DFL	N100	B05	49,722,910	1	45.77	11.8	Plant development

*Chr* chromosome, *NSNPs* number of associated significant SNPs, *Dis. (kb)* distance of annotated gene from nearest significant associated SNPs with the trait, *R*^*2*^ phenotypic variation explained in percentage.

Four floral meristem identity genes: *AGL24 (AGAMOUS-LIKE 24)–AT4G24540, AP1 (APETALA1)–AT1G69120, AP2 (APETALA2)–AT4G36920* and *JMJ14 (JUMONJI 14)–AT4G20400* were predicted*.* SNPs associated with these genes explained 2.63–13.19 percent of the phenotypic variation observed for flowering and derived traits. SNPs annotating three copies of *AGL24-AT4G24540* were located in genomic regions on A02 (34,934,199–34,934,540), A03 (33,169,034–33,177,336) and B02 (59,088,464; 64,496,077–64,499,514). *AP1-AT1G69120* was envisaged close to the genomic region harboring 13 SNPs associated for HTU and PTU. This gene was envisaged close (0.04 kb) to the peak SNP on the chromosome A02. Other copy of the same gene was predicted on chromosome B02. *AP2-AT4G36920* and *JMJ14-AT4G20400* were identified for DFL on chromosomes B02 and B03 respectively. GWAS led to the prediction of 15 genes involved with flowering regulation. Majority of these MTA's were associated with A-genome. *KHZ1-KHZ2-AT5G06770, FLOR1-AT3G12145, FLC-AT5G10140* and *FLM (FLOWERING LOCUS M)—AT1G77080* were envisaged on chromosome A02. *KHZ1-KHZ2-AT5G06770* and *FLOR1-AT3G12145* appeared to influence variation for GDD, HTU and PTU. These genes were envisaged at the distances of 2.56 and 2.99 kb from the respective peak SNPs. *FLOWERING LOCUS C (FLC)-AT5G10140* was predicted at a distance of 10.61 kb from the SNP A02_1762477. *FLM-AT1G77080* was annotated in vicinity of 28 SNPs associated with DFL, GDD, HTU and PTU. A distance of 0.82 kb separated *FLM* from the peak SNP. *FLOWERING LOCUS D (FLD)-AT3G10390* appeared close (0.94 kb) to the SNPs located on chromosome A03 (19,913,046–19,923,910). *AGL6* (*AGAMOUS-LIKE 6*) and *SOC1* (*SUPPRESSOR OF OVEREXPRESSION OF CO1*) also known as *AGL20* (*AGAMOUS-LIKE 20*) were detected on chromosome A05. Associated SNPs were detected for DCF and MRT at N75. *AT2G32950-COP1* was predicted < 2 kb away from peak SNP (N75) present on chromosome A05 (7,544,327–754,699). *AT1G68050-FKF1* (A07) could explain variation for DFI at N100. We predicted two copies of *FT (FLOWERING LOCUS T)-AT1G65480* on the chromosomes A07 and B06. These explained variation for DFL and MRT at N100. *ARP6-AT3G33520* was envisioned on chromosomes A08 for its role in the heredity of DCF. One copy each of *FVE* was recognized on chromosomes A09 and B04 at respective distances of 3.43 kb and 0.66 kb from the peak SNPs. Two copies of *AT4G00650*-*FRIGIDA (FRI)*, an important flowering regulator, were recognized on chromosomes A10 and B08. Six SNPs on B06 (8,515,215–8,524,113) were found associated at the distance of 46.85 kb from *MAF5-AT5G65080*. *HAM1 (HISTONE ACETYLTRANSFERASE OF THE MYST FAMILY 1)—AT5G64610* was also predicted at a distance of 0.61 kb from key SNP on chromosome B07. A gibberellin pathway gene, *AT3G05120-GID1A* (*GA INSENSITIVE DWARF 1A*) seemed to influence variation for DFI, DFL and DCF at N75 and N125 levels of N application. Another gibberellin pathway gene, *AT4G02780-GA1 (GA REQUIRING 1),* was recognized on chromosomes B03. *ATGA3ox3* (*ARABIDOPSIS THALIANA GIBBERELLIN 3-OXIDASE*), also predicted on chromosome B03, was significant contributor to variation observed for DFL trait at N100 level. Three genes involved in development processes of plants were envisaged: *AT5G56860-GNC (GATA NITRATE-INDUCIBLE CARBON-METABOLISM-INVOLVED), ATBPC2-AT1G14685, FAF2* (*FANTASTIC FOUR 2*)*–AT1G03170* and *ATJ3 (ARABIDOPSIS THALIANA DNAJ HOMOLOGUE 3)-AT3G44110*. The chlorophyll biosynthetic gene *AT5G56860-GNC* was significant for PH on A03 (5,286,505–5,290,395) at N75 and N100 levels of N application. The same gene was detected for DFI trait close to the SNP B08_14118234 (5.71 kb) at N100. Only one SNP, B03_115277 was identified 0.06 kb away from *FAF2* or *FTM5* (*FLORAL TRANSITION AT THE MERISTEM 5*) which is responsible for regulating shoot meristem size in *A. thaliana.* Similarly, *ATJ3-AT3G44110* was detected at B05. We also estimated LD blocks for adjacent SNPs present within 1 Mb of the predicted genes (Table 3). 43 significant SNP blocks were identified. Of these, 41 (95%) were located within the LD blocks. SNPs within LD block had LD (*r*^*2*^) values > 0.20. Size of these LD blocks ranged from 0.01 to 225.16 kb. Longest LD block (225.16 kb) was identified on chromosome A02 from 34,557,898 to 34,783,055 bp for gene *AP1-AT1G69120*. It also included the maximum number of SNPs (366). In contrast, numbers of significant SNPs (78) were located in terminal region of chromosome A02 from 1,202,251 to 1,217,865 bp within the LD block of 23.75 kb.

**Table 3:** LD block estimation for SNPs within 1 Mb regions of predicted genes associated with flowering and plant height traits.

Chr	Predicted gene	GWAS-SNPs		LD block estimation			
		SNP block	NASNPs	LD block		Block size (kb)	NSNPB
**A02**	*KHZ1-KHZ2-AT5G06770*	1,202,251–1,217,865	78	1,202,251–1,226,004		23.75	116
	*FLOR1-AT3G12145*	1,202,251–1,217,865	78	1,202,251–1,226,004		23.75	116
	*FLC-AT5G10140*	1,762,477	1	1,751,245–1,788,639		37.40	48
	*AP1-AT1G69120*	34,755,893–34,758,537	5	34,557,898–34,783,055		225.16	366
	*AGL24-AT4G24540*	34,934,199–34,934,540	6	34,934,048–34,942,039		7.99	44
	*CDF1-AT5G62430*	36,472,064–36,472,185	6	36,461,389–36,486,270		24.88	23
		36,486,270–36,487,130	5	36,461,389–36,486,270		24.88	23
				36,486,488–36,495,138		8.65	27
	*FLM-AT1G77080*	37,090,239–37,093,082	23	37,086,932–37,091,727		4.80	46
				37,091,896–37,133,361		41.47	81
		37,133,361–37,136,964	5	37,135,994–37,136,964		0.97	4
	*FLD-AT3G10390*	19,913,046–19,923,910	8	19,887,999–19,956,139		68.14	51
	*AGL24-AT4G24540*	33,169,034–33,177,336	4	33,168,278–33,179,337		11.06	19
**A05**	*CKB4-AT2G44680*	2,937,409–2,945,567	5	2,736,655–2,937,429		200.78	20
				2,944,442–2,952,048		7.61	8
	*AGL6-AT2G45650*	3,354,215	1	3,301,121–3,356,339		55.22	11
	*SOC1-AT2G45660*	3,354,890–3,357,582	3	3,301,121–3,356,339		55.22	11
				3,357,582–3,357,639		0.06	3
	*COP1-AT2G32950*	7,544,327–7,546,996	24	7,437,216–7,445,302		8.09	14
				7,451,045–7,480,422		29.38	19
	*GID1A-AT3G05120*	30,987,755–30,990,279	3	30,987,755–30,990,279		2.53	3
**A07**	*FKF1-AT1G68050*	1,786,248	1	1,764,823–1,822,282		57.46	54
	*FT-AT1G65480*	33,488,659	1	33,403,301–33,535,523		132.22	7
**A08**	*ARP6-AT3G33520*	52,619–52,842	10	12,259–146,533		134.28	156
	*FD-AT4G35900*	18,952,302–18,955,620	10	18,952,302–18,952,343		0.04	2
				18,952,388–18,952,521		0.13	2
				18,954,147–18,954,150		0.01	2
				18,954,994–18,966,049		11.06	17
**A09**	*FVE-AT2G19520*	10,079,474	1	10,075,137–10,091,939		16.80	51
	*GI-AT1G22770*	39,780,608–39,780,959	3	39,761,855–39,825,243		63.39	97
**A10**	*FRI-AT4G00650*	6,054,217–6,054,243	2	6,016,556–6,054,243		37.69	63
**B02**	*AP1-AT1G69120*	59,351,856–59,353,415	8	59,351,856–59,352,264		0.41	7
				59,352,392–59,353,135		0.74	6
				59,353,276–59,353,285		0.01	3
	*GID1A-AT3G05120*	62,297,219–62,297,220	2	62,280,062–62,302,328		22.27	119
	*AGL24-AT4G24540*	64,496,077–64,496,528	3	64,487,988–64,555,102		67.12	77
		64,497,529–64,499,514	3	64,487,988–64,555,102		67.12	77
	*AP2-AT4G36920*	66,542,016–66,546,535	6	66,496,080–66,562,989		66.91	105
**B03**	*FAF2-AT1G03170*	115,277	1	115,081–254,900		139.82	80
	*CRY1-AT4G08920*	338,948–346,586	15	289,286–343,540		54.26	72
	*JMJ14-AT4G20400*	10,133,662–10,134,104	2	10,129,276–10,153,357		24.08	67
**B04**	*FVE-AT2G19520*	16,336,556–16,341,785	7	16,258,914–16,407,739		148.83	74
**B05**	*ATJ3-AT3G44110*	49,722,910	1	49,689,123–49,735,541		46.42	46
**B06**	*FT-AT1G65480*	6,716,803–6,718,200	5	6,716,803–6,736,028		19.23	39
	*MAF5-AT5G65080*	8,515,215–8,524,113	6	8,481,230–8,519,496		38.27	19
				8,524,113–8,650,692		126.58	135
**B07**	*HAM1-AT5G64610*	11,563,802–11,564,171	5	11,464,372–11,564,320		99.95	94
**B08**	*CO-AT5G15840*	9,748,710–9,748,737	2	9,743,391–9,767,625		24.24	43
	*GNC-AT5G56860*	14,118,234	1	14,064,467–14,154,003		89.54	84
	*FRI-AT4G00650*	16,066,635	1	16,065,157–16,070,876		5.72	18

*Chr* chromosome, *GWAS-SNPs* significant SNPs identified in GWAS study, *SNP block* coordinates of identified significant SNPs in base pair, *NASNPs *number of associated SNPs, *LD block* estimated LD blocks for SNPs within 1 Mb region, *block size (kb)* distanced spanned by block in kb, *NSNPB* number of SNPs in LD block.

## Discussion

Days to flowering, maturity and plant height have been the major targets of selection during domestication due to their importance for the reproductive success, uniform ripening and ease of harvesting. Breeding for these traits is important even now as new cultivars must fit into newly emerging cropping systems and geographic niches. Optimum transition to flowering is also important to correct climate change-induced “phenological mismatches” that are creeping into many crops and their current ecosystems. However, phenological alterations are difficult to accomplish due to the complex genetics and strong genotype × environmental interactions. These hinder selection gains. N is an important external cue as it is both a plant nutrient and a signalling molecule^33^. N availability affects plant phenotypes by genome-wide changes in the expression pattern(s) of the genes associated with different metabolic processes^34–36^. Testing of genotypes at three levels of N application in the current studies produced highly significant G × Y, G × N and G × N × Y interactions. This was an expected as phenotypic expression of a genotype is only one among many phenotypic manifestations’ realizable under different environments. Although the test genotypes varied for their individual responses, most flowered and matured earlier at N75 in comparison to N100 and N125. Lower N availability induces early flowering^37–39^, while high N delays it^40^. Genotype-specific flowering time variations in response to the applied N have been observed earlier in *A. thaliana*^41^. Population structure and kinship analysis allowed clustering of germplasm into three broad groups. Most of the resynthesized and exotic (largely east European) genotypes fell in to the groups distinct from the one harbouring indigenous *B. juncea* lines with some admixing. This kind of population structure suggested an independent evolution of Indian and east European genotypes. Admixing may have resulted from extensive inter-varietal hybridizations practised by earlier and present-day plant breeders. We used GWAS, an LD based method, to investigate trait genetics. The resolution with which a QTL can be mapped depends upon the speed of LD decay, which is the outcome of multiple genetic recombination’s across the genome^42,43^. Mustard is a self-pollinated crop, with varying amount of cross pollination. LD was distributed unevenly among chromosomes and two sub-genomes in our test panel. It decayed to approximately 0.21 for SNPs at a distance of 0.20 Mb. Mean *r*^*2*^ also dropped below 0.1 beyond 1 Mb inter-marker distance. A rapid LD decay in our association panel confirmed its suitability for conducting GWAS. There is no past report about LD decay estimated using a large number of biallelic markers in *B. juncea.* However, present outcomes are consistent with those in related allotetraploids of *B. napus*^44^ and *B. carinata*^45^.

A large number of SNPs were identified as significant for association with flowering, maturity, plant height and derived traits. These SNPs differed for their chromosome locations and effects over N-levels. Identified SNPs explained significant proportions of the phenotypic variations recorded for the evaluated traits. In silico annotation within a threshold window of genes within 100 kb (50-kb upstream and 50-kb downstream) of the peak SNP allowed prediction of 30 candidate genes belonging to light perception, circadian pathway, floral meristem identity, flowering regulation, gibberellic acid pathways and plant development. This threshold window was selected on the basis of low levels of overall LD (200 kb) in our association panel. Majority of identified genes were predicted from the associations found significant at N75 and N100. Nitrate availability is known to alter the expression of major genes belonging to photoperiod (e.g. *CO*, *CRY1*) and circadian pathways in *Arabidopsis*^46^. Photoperiod pathway also interacts with the gibberellin acid and autonomous pathways to modulate nitrate-regulated floral transition^47^. Nitrates repress flowering time via gibberellin pathway^48^. Loss of function or overexpression of many genes in these pathways cause major flowering time changes^46,47,49^. The annotated genes included more than one copy each of six important flowering genes (*AGL24*, *AP1*, *FVE*, *FRI*, *GIDIA* and *GNC*). Multiple copies of each of the flowering genes are expected as *Brassicas* are ancient polyploids^50^. The resultant functional redundancy can impact phenotypic diversity if multiple gene copies act in an additive or dosage-dependent manner^51^. *FD*, predicted on chromosome A08, encodes a *bZIP* transcription factor which is a positive regulator of flowering. It promotes flowering following interaction with *FT*^52^. *CRY1* was detected on chromosome B03 at all three levels of N application. It is one of the two key factors involved in N-regulated flowering time control in *Arabidopsis thaliana*, other being ferredoxin-NADP^+^-oxidoreductase. Loss-of-function mutants of *CRY1* are insensitive to N availability^46^. *CRY1* also acts through N signal pathway to regulate flowering output genes *CO* and *GI*. These are upregulated if plants are grown under limiting nitrate conditions^46^. *CO* was envisioned on chromosome B08. Overexpression of *CO* and *FLC* eliminates the influence of nitrates on flowering^47^. Circadian clock controlled flowering pathway is the timekeeper of photoperiodism where *GI* promotes the expression of flowering-time genes, *CO* and *FT*^53^ as well. *AP1* and *AP2* were predicted close to the SNPs identified significant at N75 on chromosomes A02 and B02 respectively. Two more copies of *AP1* were envisaged on the chromosomes A09 and B02 at N100. *AP1*, *AP2* and *AGL24* regulate initial stages of flower development and redundantly act to control floral meristem identity^54^. *AGL24* is upregulated in the inflorescence apex during floral transition^55^. Three copies of this gene were predicted on chromosomes A02, A03, and B02. Associated SNPs were identified at N100 and N125 but not under N deficiency (N75). Another gene *JMJ14* was predicted on chromosome A07, in vicinity of SNPs found significant at N125. It encodes a histone *H3K4* demethylase which prevents early flowering through repression of the floral integrators *FT*, *AP1*, *SOC1* and *LFY* during the vegetative growth phase^56^. *FT*, predicted on A07 and B06, is the core of photoperiodic flowering pathway and it is present downstream of the *GI* and *CO*^57^. *SOC1*/*AGL20* was predicted on the chromosome A05 for the traits DCF and MRT under N deficient conditions. It integrates signals from the photoperiod, vernalization and autonomous floral induction pathways^58^. *SOC1* is upregulated under N deficiency^38^.

We predicted *FLC1* (A02) and *FT* (A07, B06) at N100. *FLC* is important for the initiation timing of flowering. Three *FLC* homologs are known in *Brassica*^50,59^. It represses *FT and SOC1,* to prevent the conversion of apical meristem into the reproductive structures^60^*.* Significant downregulation of *FLC* and upregulation of *FT*, *LFY*, and *AP1* has been reported under N deficient conditions^37^. Another predicted gene *ATJ3* (B03) acts downstream of various floral pathways and mediates the transcriptional regulation of *FT* and *SOC1,* during switch to flowering via a known flowering repressor *SVP*^61^. *FRI* up regulates the expression of *FLC* to accelerate the transition to flowering after the vernalization^62^. A copy each of this gene was identified on chromosomes A10 and B08 under N75 and N125. *FVE* is a key regulator of the autonomous pathway that reduces *FLC* expression. A similar gene *ARP6* that encodes actin-related protein, a putative component of a chromatin-remodelling complex, is required for both histone acetylation and methylation of the *FLC* chromatin in *Arabidopsis* and controls its expression^63^. *HAM1,* identified on B07, affects the flowering time by epigenetic modification of *FLC*^64^. Other flowering genes, *FLOR1*, *FLR1* and *FTM4* were identified on chromosome A02. *FLOR1*, predicted under high N conditions, interacts with the MADS box transcription factor *AGAMOUS* to delay flowering. Its role is partially redundant with *SOC1* and *FUL*^65^. *MAF1* or *FLM*, which is related to *FLC* was identified on A02. It acts as the negative regulator of flowering. *FLD* located on A03, plays a key role in regulating the reproductive competence of the shoot by repressing *FLC*^66^. *AGL6*, present on A05, act as a floral promoter with roles in inhibition of *FLC/MAF* genes and promotion of *FT*^67^. *COP1* modulates the circadian rhythm and flowering. It encodes a RING-finger E3 ubiquitin ligase which along with SUPPRESSOR of *phyA-105* (SPA) proteins, represses photoperiodic flowering by regulating proteasome-mediated degradation of *CONSTANS (CO)*^68^. *MAF5/*AGL68 also regulates the flowering time during the vernalization. Nitrates are known to modulate the expression of *TEM1*. This gene control floral transition by repressing *FT* and GA-dependent flowering pathways by regulating *GA3ox1* and *GA3ox2*^69^. *FKF1,* a flavin-binding kelch repeat F box protein was identified on chromosome A07 under variable N levels. It is clock-controlled gene that regulates transition to flowering^70^. It is repressed by nitrate and is involved in the induction of *CO* and *FT*^71^. Mahmood et al.^72^ had identified a stable QTL for flowering traits on linkage group B06. This QTL simultaneously influenced flowering, maturity and plant height. Ramchiary et al.^30^ also reported QTLs for plant height on chromosomes, A01, A02 and B06 in *B. juncea.* GA is important for promoting flowering under non-inductive photoperiod conditions^73^. This pathway is affected by N availability. Low N increases GA concentration while high N reduces it^38^. *GA1* was envisaged on chromosomes A09 and B02 at all three levels of N application. *GID1A*, a positive regulator of flowering and a stable soluble gibberellin receptor^74^, was predicted on A05 and B02 for the traits DFI, DFL and DCF under N75 and N125. *GID1A* is a soluble gibberellin (GA) receptor and is involved in GA signaling that controls root growth, seed germination and flower development. *ATGA3ox3* identified on chromosomes A08 and B03 catalyses the final step in the synthesis of bioactive gibberellins (GAs). The gene is expressed in stamen filaments, anthers, and flower receptacles to promote their growth^75^. GATA transcription factors *GNC* (*GATA, NITRATE-INDUCIBLE CARBON-METABOLISM INVOLVED*) and *GNL*/*CGA1* (*GNC-LIKE/CYTOKININ-RESPONSIVE GATA FACTOR1*) prevent flowering by directly repressing *SOC1* expression in *Arabidopsis* gene *SOC1*^76^. One copy each of *GNC* was predicted on chromosomes A03 and B08 respectively. These may explain part of variation recorded for DFI and PH at N75 and N100. *GNC* has a major role in the chlorophyll biosynthesis and it is upregulated by N and repressed by GA signalling^77^. Plant height is a major yield component in mustard. Though plant height is positively associated with yield, tall plants are more prone to lodging, especially as they approach maturity. Lodging can strongly reduce yield and impair grain quality. Genotypes with reduced height can withstand lodging better and are more suitable for mechanical harvesting. In the present studies, 18 SNPs on A09 in the genomic region of 52,234,633–52,238,177 were significantly associated with both plant height and flowering time. As described earlier, majority of the associated SNPs were located within the LD blocks. Identification of a fairly number of SNPs in high LD is important as any SNP can be used as a tag SNP. However, the tag SNPs are required to be selected independently if associated SNPs are present in more than one group with little intergroup LD.

Summarising, we used multiple phenotypic data (DFI, DFL, DCF, MRT, GDD, HTU, PTU, and PH) and multilayer evaluation to detect a large number of MTA's showing low to moderate contributions to respective trait variations. Most of the associated SNPs were distributed around key flowering genes: *CRY1, CO, SOC1/AGL20, AGL6, AGL24, GI, AP1, AP2, FLC, FT, ARP6, FVE, ATGA3OX3, FLOR1, GID1A,* etc. Majority of these were located within a distance of 25 kb from respective peak SNPs. Broadly, our results are consistent with flowering QTLs reported earlier for chromosomes A02, A03, A05, A06, A08, A09, B02, B03, B06, B07 and B08 in *B. juncea* ^24,30,32,72^. The information on marker-trait associations and validation of candidate genes predicted during our studies may promote marker aid breeding (MAB) in Indian mustard.

## Methods

### Plant material and phenotyping

Association panel comprised 92 germplasm lines maintained for seven generations of selfing as per single seed descent method. The panel included advanced breeding lines (BL) or cultivars (CV), germplasm from east Europe, Australia (EM), derived resynthesized *B. juncea* (DJ), resynthesized determinate mustard (DTM), introgression lines (IL). All the genotypes included in the association panel had very high (> 90%) and euploid chromosome number expected for *B. juncea* (2n = 36;AABB). The details information about the genotypes included in the association panel is available elsewhere^78^. The trials were conducted at the farms of Punjab Agricultural University at Ludhiana (30.9010° N, 75.8573° E) for two crop seasons (2015–16 and 2016–17, hereafter designated as year 1 and year 2 respectively) in alpha lattice design with two replications and three levels of nitrogen (N) {low dose (N75, with added N @ 75 kg/ha), standard dose (N100, with added N @100 kg/ha) and high dose (N125, with added N @ 125 kg/ha.)}. Urea (46% N) was applied in two splits, half at the time of seeding and the second half, 22 days after sowing. We raised each genotype in a plot of four rows, each row measuring two meters in length. The rows were spaced 30 cm apart. The crop was sown during second fortnight of October and it was harvested during second week of April every year. The crop was flood irrigated at three times during the growing season. Standard agronomic practices were followed for the other crop inputs. Days to flowering were counted three times: between sowing of the crop till the commencement of flowering (DFI), when 50% of plants had started to flower (DFL) and culmination of flowering (DCF). We counted days from seeding when crop had attained physiological maturity (DM). Physiological maturity was reached when 90 percent of pods had turned light brown. Plant height (PH) was measured in centimetres from base of the plant till the tip of the main shoot at maturity. We also estimated growing degree days (GDD), referred to as sum of mean daily temperatures (°C) above a defined temperature threshold of 5°C^79^.

$${\text{GDD }}\left( {^\circ {\text{C day}}} \right) \, = \, \left\{ {\left( {{\text{T}}_{{{\text{maximum}}}} + {\text{ T}}_{{{\text{minimum}}}} } \right)/{2}} \right\} \, - {\text{ T}}_{{{\text{base}}}} .$$

The growing degree day value for a given day was considered zero, if the average temperature fell below the temperature threshold or the base temperature (5 °C).

We computed Helio-thermal unit (HTU) and photo-thermal units (PTU). HTU is the product of GDD and corresponding actual sunshine hours of that day and PTU is the product of GDD and corresponding day length^80^.

$$\begin{gathered} {\text{PTU }}\left( {^\circ {\text{C day hours}}} \right) \, = {\text{ GDD }} \times {\text{ day length}}. \hfill \\ {\text{HTU }}\left( {^\circ {\text{C day hours}}} \right) \, = {\text{ GDD }} \times {\text{ Actual bright sunshine hours}}. \hfill \\ \end{gathered}$$

GDD, PTU and HTT values were computed at the initiation of flowering.

The agro-meteorological data for everyday temperature, sunshine hours and day length were sourced from the Department of Climate Change & Agricultural Meteorology, Punjab Agricultural University, Ludhiana, Punjab, India.

### Analysis of phenotypic variation

Analysis of variance (ANOVA) was implemented to test significance of variation owing to genotypes, nitrogen levels, crop seasons and their interactions. GLM (generalized linear model) was implemented in alpha lattice design^81^. The analysis was carried out using SAS version 9.4. Basic equation was:

$$Y_{ijk} = \, \mu \, + \, t_{i} + \, r_{j} + \, b_{jk} + \, e_{ijk}$$

where *y*_*ijk*_ denotes values for the observed traits for *i*th treatment in the *k*-the blocks within *j*-th replicate (superblocks), *t*_i_ is the fixed effects of the *i*th treatment (i = 1,2,….,t); *r*_*j*_ is the effects of *j*th replicate (superblocks) (j = 1,2,….,r); *b*_*jk*_ is the effects of the *k*th incomplete blocks within the *j*th replicate (k = 1,2,…s) and *e*_*ijk*_ is an experiment error associated with the observation of the *i*th treatment in the *k*-th incomplete block within *j*-th complete replicate.

### Correlation analysis

Pearson correlation coefficients were first estimated and these were plotted by R-package “corrplot”.

### SNP genotyping and genome wide association analysis (GWAS)

The diversity set was genotyped by sequencing (GBS)^82^. Total genomic DNA was extracted from young leaves of 92 genotypes using a standard CTAB method with minor modifications. DNA samples were quantified by visual comparison to λ-DNA standards on ethidium bromide-stained agarose gels. The purity and concentration of the samples was checked with spectrophotometer readings at 260 and 280 nms. High quality DNA samples were genotyped by sequencing (GBS) on the ILLUMINA HiSeq platform, which was outsourced to Novogene (HK) Company Limited, Hongkong. Bioinformatic analyses were conducted with publicly available software’s. The reference genome of *B. juncea* v1.5 was used for alignment of whole genome sequence (25x) of a commercial *B. juncea* genotype, PBR357 with the software "bowtie2". SNP calling was implemented in NGSEP-GBS pipeline^83^. Total SNPs were replaced in *B. juncea* genome reference using a perl script, pseudomaker.pl implemented in the SEG-Map to construct mock-up pseudomolecule reference. All 92 inbred lines of the diversity set were then aligned on pseudomolecule genome reference and SNPs were identified with NGSEP-GBS pipeline. We improved the SNP density of through imputation, using a mustard reference SNP set with 570,764 high quality SNPs. This reference set was developed through re-sequencing (10–12 Χ genome coverage) of a mustard germplasm core set comprising 96 genotypes. Software minimac2 was used for imputation^84^. A total 406,888 SNPs were retained for GWAS after filtration. The filtration parameters were MAF > 0.05 and maximum heterozygosity < 10%. A sub-set of 66,835 SNP set, available with stringent filtration (MAF > 0.10) and prior to imputation, was used to determine population structure and linkage disequilibrium. Software’s PGDSpider v2.1.1.3 and ‘GAPIT’^85^ were used for converting SNP genotypes into the structure and numeric formats, respectively.

### Population structure and linkage disequilibrium

Population structure was developed with the software STRUCTURE 2.3.4. and 1–10 subgroups (*K*). Ten runs for each K were performed by a model assuming admixture and correlated allele frequencies. The run length was 10,000 period followed by Markov Chain Monte Carlo (MCMC) repetitions, set to 1,00,000 replications. The optimum number of subgroups (K) was selected on the basis of the log probabilities LnP(D) and ad hoc statistic ∆K method^86^. Second order rate of change of the likelihood function for K was determined using Structure Harvester^87^. Linkage disequilibrium (LD) was estimated with TASSEL v5.0^88^ based on squared allele frequency correlations (*r*^2^) between all pairs of SNP markers.

### Association mapping

Phenotypic data was normalized by Johnson transformation as implemented in Minitab v16.0. We used software GAPIT3^89^ for trait-SNP association analysis. Q–Q plots aided the recognition of best GWAS model among five algorithms (GLM, MLM, FarmCPU, MLMM and Blink) implemented in GAPIT3. SNP-trait associations were classified as significant on the basis of an arbitrarily chosen threshold of –log10 (*P*) ≥ 3.0. Bonferroni threshold or a P value adjusted by a false discovery rate of 0.05 was considered too restrictive stringent. Pairwise LD values for the trait associated SNPs were estimated within 1 Mb window using the software Plink1.09 (https://www.cog-genomics.org/plink2) with parameters: -noweb -all -blocks -no-pheno-req -ld-window-kb 1000.

### In silico analysis for candidate gene identification

50-kb flanking regions surrounding the peak SNPs were scanned to predict candidate genes. The predicted gene and its orthologous sequences were then annotated by BLAST analysis against the *Arabidopsis thaliana* database using Blast2GO v5.0 tool^90^. Functions of the possible candidate genes were checked in the literature to determine their relevance for the trait in question.

## Supplementary Information

Supplementary Figures.

Supplementary Tables.

## Data Availability

Sequencing data for the test genotypes is available at National Center for Biotechnology Information (https://dataview.ncbi.nlm.nih.gov/object/PRJNA639209?reviewer=gb2flbo53a3kcv2ts2bfslphhc) as Bio-Project PRJNA639209. Supply of germplasm resources will require approval of Biodiversity Authority of India.
