# Supplementary material for: Genome wide association analyses to understand genetic basis of flowering and plant height under three levels of nitrogen application in Brassica juncea (L.) Czern & Coss
Source: Sci Rep. 2021 Feb 19;11:4278. doi: 10.1038/s41598-021-83689-w (PMC7896068; doi:10.1038/s41598-021-83689-w)
Supplement: Supplementary file 1 — Supplementary Figures. [file 41598_2021_83689_MOESM1_ESM.pdf]

Genome wide association analyses to understand genetic basis of flowering and plant height under three levels of nitrogen application in *Brassica juncea* (L.) Czern & Coss.

Javed Akhatar, Anna Goyal, Navneet Kaur, Chhaya Atri, Meenakshi Mittal, Mohini Prabha Singh, Rimaljeet Kaur, Indu Rialch and Surinder S. Banga*

Department of Plant Breeding and Genetics, Punjab Agricultural University, Ludhiana -141004, Punjab, India.

***Corresponding Author:** Surinder S. Banga; E-mail address: nppbg@pau.edu, Contact No. +91-94633-19390; ORCID ID: https://orcid.org/0000-0001-8209-7341

This file contains Supplementary Table 1.

Supplementary Table S1. Analyses of variance (ANOVA) for the flowering traits studied at three different N – Level (N75, N100 and N125).

Trait		Source of Variation											
		**Treatment**	**Year**	**Replication**	**Block (Replication)**	**N-level**	**Treatment × N-level**	**Treatment × Year**	**Replication × N-level**	**Replication × Year**	**N-level × Year**	**Treatment × N-level × Year**	**Error**
	**Year**	**DF 91**	**1**	**1**	**6**	**2**	**182**	**91**	**2**	**1**	**2**	**182**	**Year1&2:267 Year P:542**
**DFI**	1	99.79**	--	1,206.47**	107.57	1,078.69**	39.04	--	878.34**	--	--	--	55.20
	2	115.29**	--	79.89	174.21*	1,393.68**	56.96	--	109.02	--	--	--	76.27
	P	114.64**	1,139.30**	953.64**	121.13	2,197.49**	53.06	102.92**	799.58**	332.72*	274.88*	42.95	67.23
**DFL**	1	175.43**	--	739.87**	185.73*	349.12**	39.20	--	1,013.27**	--	--	--	74.27
	2	171.98**	--	32.05	174.80*	398.98**	48.30	--	21.87	--	--	--	77.90
	P	200.82**	1,293.35**	231.98	212.71*	561.39**	43.70	153.10**	588.98**	539.94**	186.71*	43.81	78.24
**DCF**	1	104.82**	--	3,006.73**	106.06*	6,284.91**	28.94	--	2,232.82**	--	--	--	48.54
	2	174.12*	--	1,299.65**	107.68*	1,544.25**	96.63	--	336.16	--	--	--	120.24
	P	137.23**	10,051.17**	176.40	150.18	3,870.72**	62.69	147.11**	929.92**	4,129.99**	3,958.44**	62.88	89.90
**MRT**	1	45.90**	--	526.31**	55.74	5,122.32**	19.52	--	35.18	--	--	--	5.46
	2	58.13	--	597.11*	55.81	11,232.88**	74.63	--	36.73	--	--	--	104.74
	P	51.24	23,091.95	1,122.30**	59.41	15,699.68**	53.25	50.53	8.05	1.12	655.51**	40.90	67.12
**GDD**	1	4,456.87**	--	63,572.46**	14,160.63**	38,775.28**	1,483.49	--	41,170.12**	--	--	--	2377.30
	2	6,034.82**	--	0.05	19,876.43**	64,997.70**	2,402.23	--	12,888.40*	--	--	--	3713.99
	P	10,190.52**	72,546.07**	31,778.72**	33,153.61**	93,843.78**	3,502.39	279.80	47,663.92**	31,862.47**	10,006.60*	385.02	3036.06
**HTU**	1	472,098.64**	--	6,694,443.25**	1,488,920.99**	4,096,496.84**	158,337.15	--	43,32,022.59**	--	--	--	252920.10
	2	639,239.02**	--	377.85	2,102,926.68**	6,913,353.69**	2,57,512.88	--	13,90,673.17*	--	--	--	395854.10
	P	1,078,223.92**	26,324,422.14**	3,297,137.24**	3,494,938.99**	9,937,696.96**	374,389.56	30,166.10	5,054,151.52**	3,397,621.85**	1,072,241.41*	41,468.12	323143.6
**PTU**	1	47,147.34**	--	331,623.24**	98,730.94**	2,63,861.98**	23,995.24	--	2,01,260.57**	--	--	--	31380.54
	2	104,090.82**	--	5,297.28	355,624.68**	1,051,019.08**	45,237.19	--	2,18,005.65*	--	--	--	67643.37
	P	136,387.84**	3,704,300.27**	126,594.99	412,123.44**	1,081,238.71**	57,628.95	13,536.28	409,977.30**	210,368.60*	233,732.95**	11,604.44	49283.73
**PH**	1	639.62**	--	29,423.82**	1,088.05*	12,688.01**	304.47	--	8,395.28**	--	--	--	394.06
	2	697.33**	--	1,335.81*	405.14	6,208.09**	164.38	--	893.43	--	--	--	300.34
	P	653.03**	20,667.19**	21,649.15**	1,071.06**	17,210.75**	243.30	690.70**	3,815.27**	9,110.48**	1,685.35*	225.55	366.94

DFI - Days to initiation of flowering, **DFL** - Days to fifty percent of flowering, **DCF** - Days to complete flowering, **MRT** - Days to maturity, **GDD** - Growing degree days, **PTU** - Photo thermal units, **HTU** - Helio Thermal units and **PH** - Plant height; *, ** Significant at *P*< 0.05 and 0.01, respectively
