# Supplementary material for: Genome wide association analyses to understand genetic basis of flowering and plant height under three levels of nitrogen application in Brassica juncea (L.) Czern & Coss
Source: Sci Rep. 2021 Feb 19;11:4278. doi: 10.1038/s41598-021-83689-w (PMC7896068; doi:10.1038/s41598-021-83689-w)
Supplement: Supplementary file 2 — Supplementary Tables. [file 41598_2021_83689_MOESM2_ESM.pdf]

Genome wide association analyses to understand genetic basis of flowering and plant height under three levels of nitrogen application in *Brassica juncea* (L.) Czern & Coss.

Javed Akhatar, Anna Goyal, Navneet Kaur, Chhaya Atri, Meenakshi Mittal, Mohini Prabha Singh, Rimaljeet Kaur, Indu Rialch and Surinder S. Banga*

Department of Plant Breeding and Genetics, Punjab Agricultural University, Ludhiana -141004, Punjab, India.

***Corresponding Author:** Surinder S. Banga; E-mail address: nppbg@pau.edu, Contact No. +91-94633-19390; ORCID ID: https://orcid.org/0000-0001-8209-7341

This file contains Supplementary Figures S1-S5.

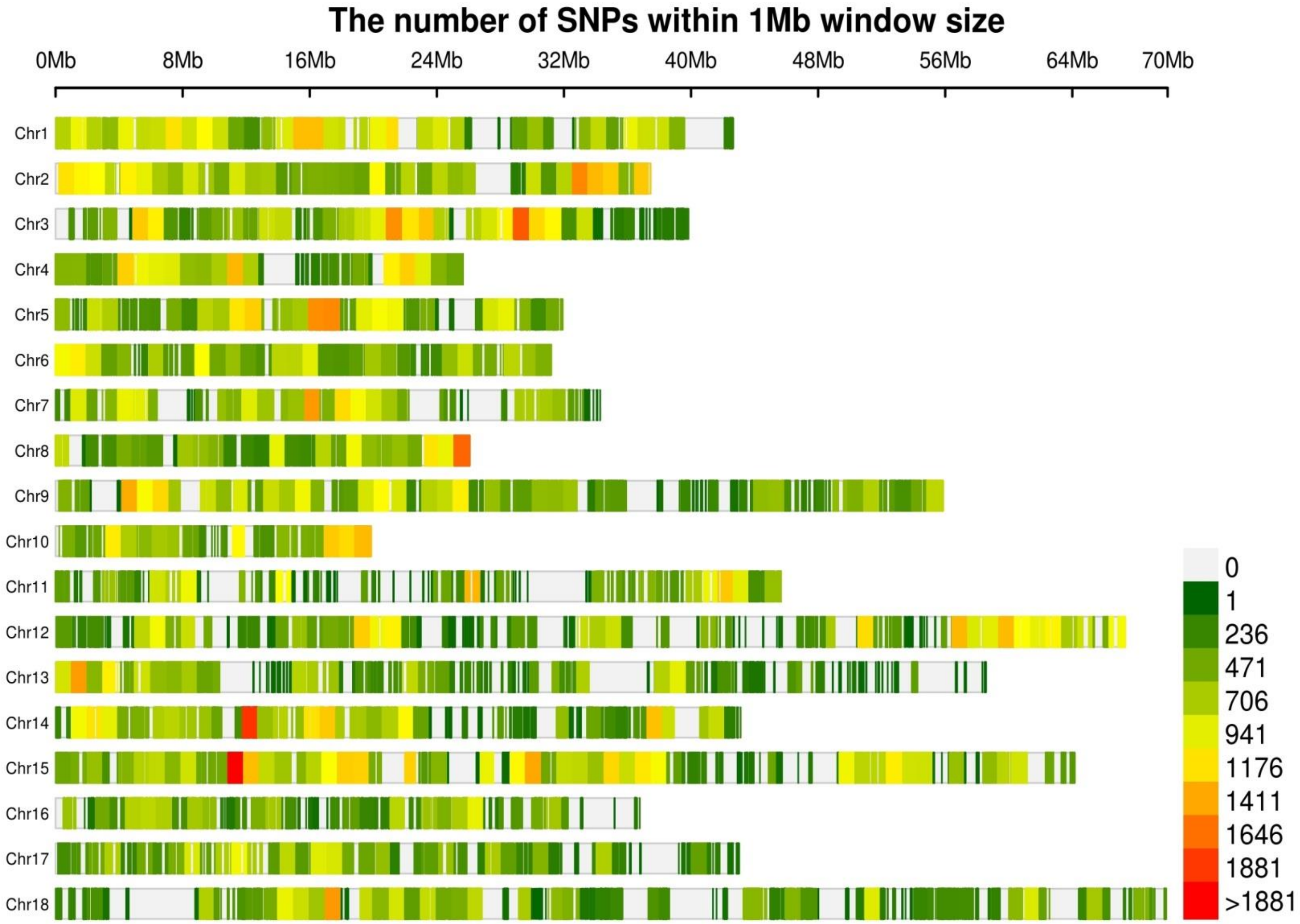

Supplementary Fig. S1. Distribution of SNPs over 18 chromosomes (Chr.1-10 A genome; Chr.11-18 B genome) of *Brassica juncea.* Number of SNPs per chromosome varied from 13,267 (Chr. A10) to 40,509 (Chr. B05). A-genome (1-10) chromosomes harboured higher number of SNPs (212,979) as compared to B-genome (11-18)chromosomes (193,909).

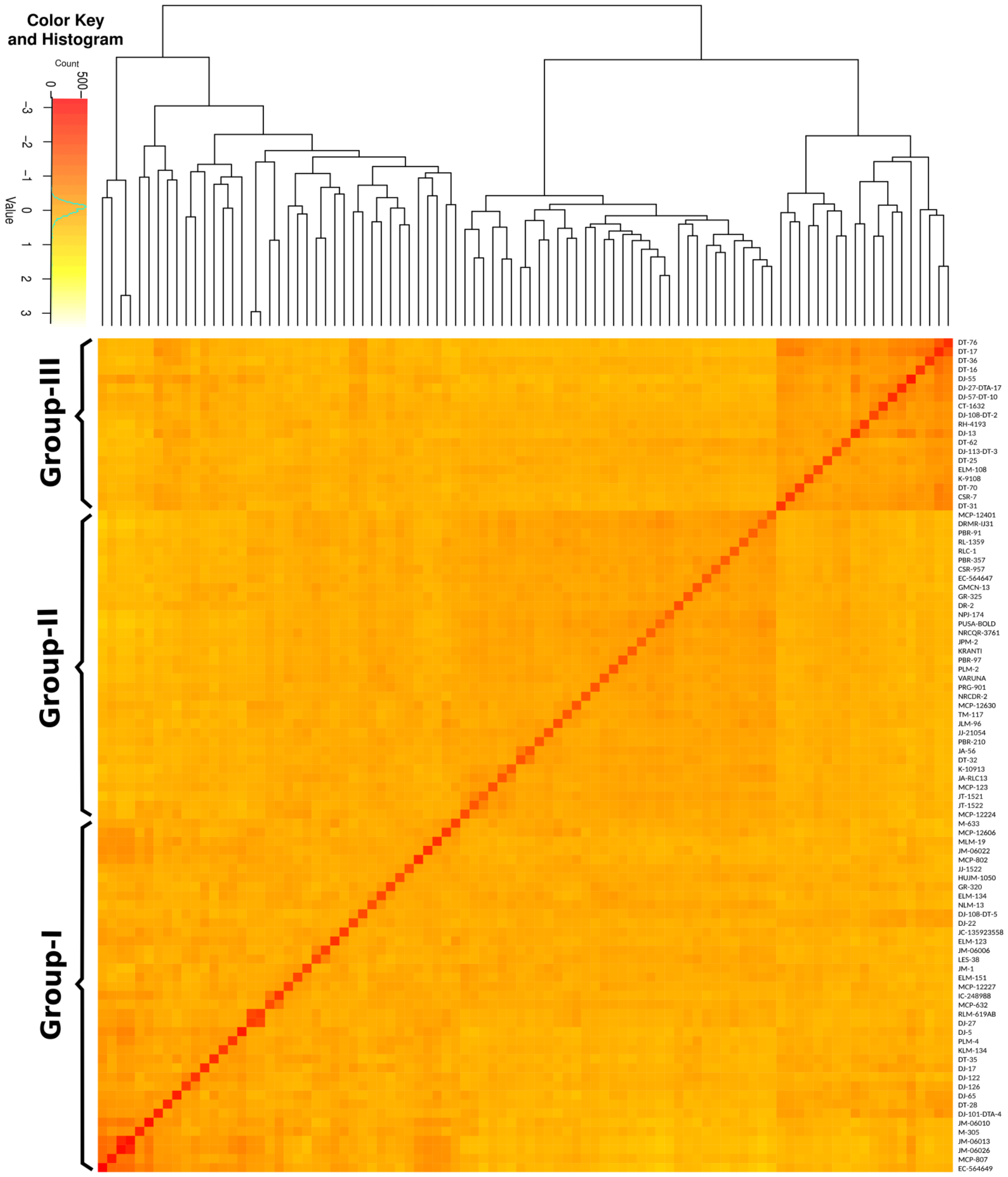

Supplementary Fig. S2. Kinship matrix suggesting existence of three broad groupings in *B. juncea* diversity panel. Maximum numbers of inbred lines fell in G1 (39) followed by G2 (34) and G3 (19).

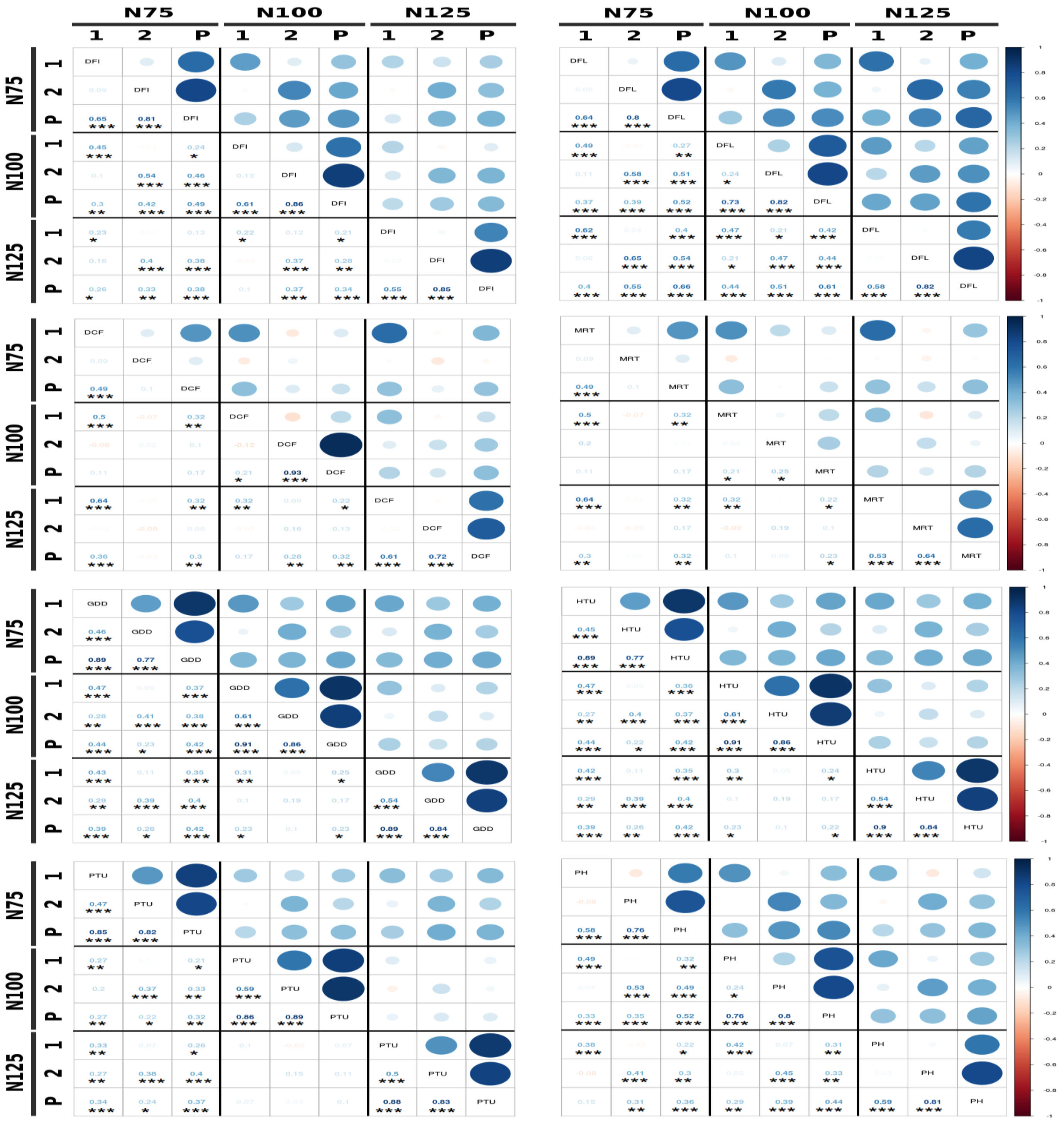

Supplementary Fig. S3. Pearson correlation coefficient analysis for life history traits in *Brassica juncea* for different years of testing (Y1, Y2 and Pooled) and doses of N application (N75, N100 and N125). Correlations are displayed as blue (positive) or red (negative) coloured circles above the diagonal for each trait. Colour intensities and circle size are proportional to the correlation coefficients. Lower diagonal for each trait plot shows actual correlation values and the levels of significance (*** = correlations with p-value < 0.001, ** = < 0.01 and * = < 0.05). A vertical colour bar on the right side of plot depicts correlation coefficients in terms of the colour intensity.

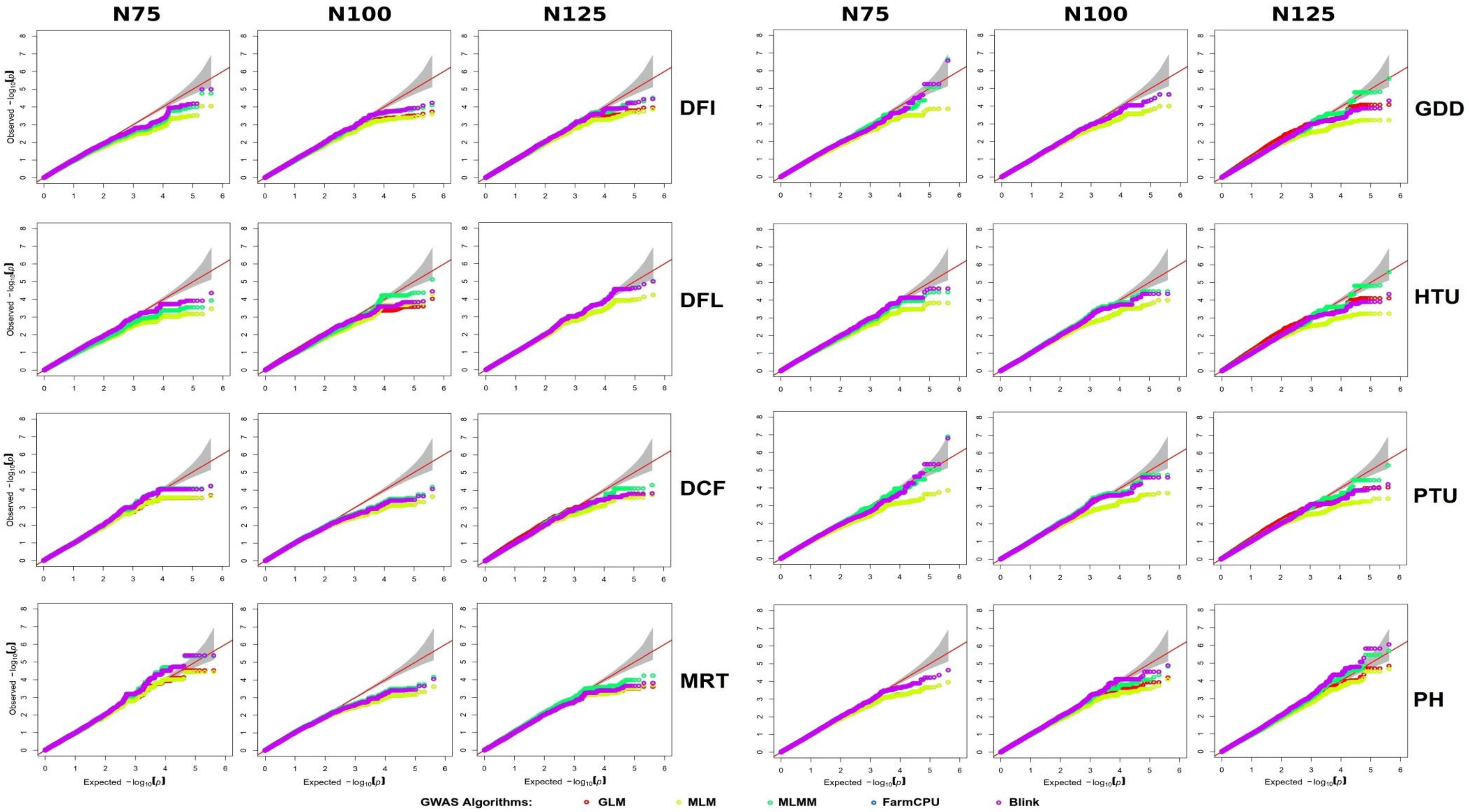

Supplementary Fig. S4. Q-Q plots of p-values for tests for multiple GWAS model with five algorithms (GLM, MLM, FarmCPU, MLMM and Blink) for flowering maturity and plant height for three N levels. p-values are depicted at the bottom of the figure.

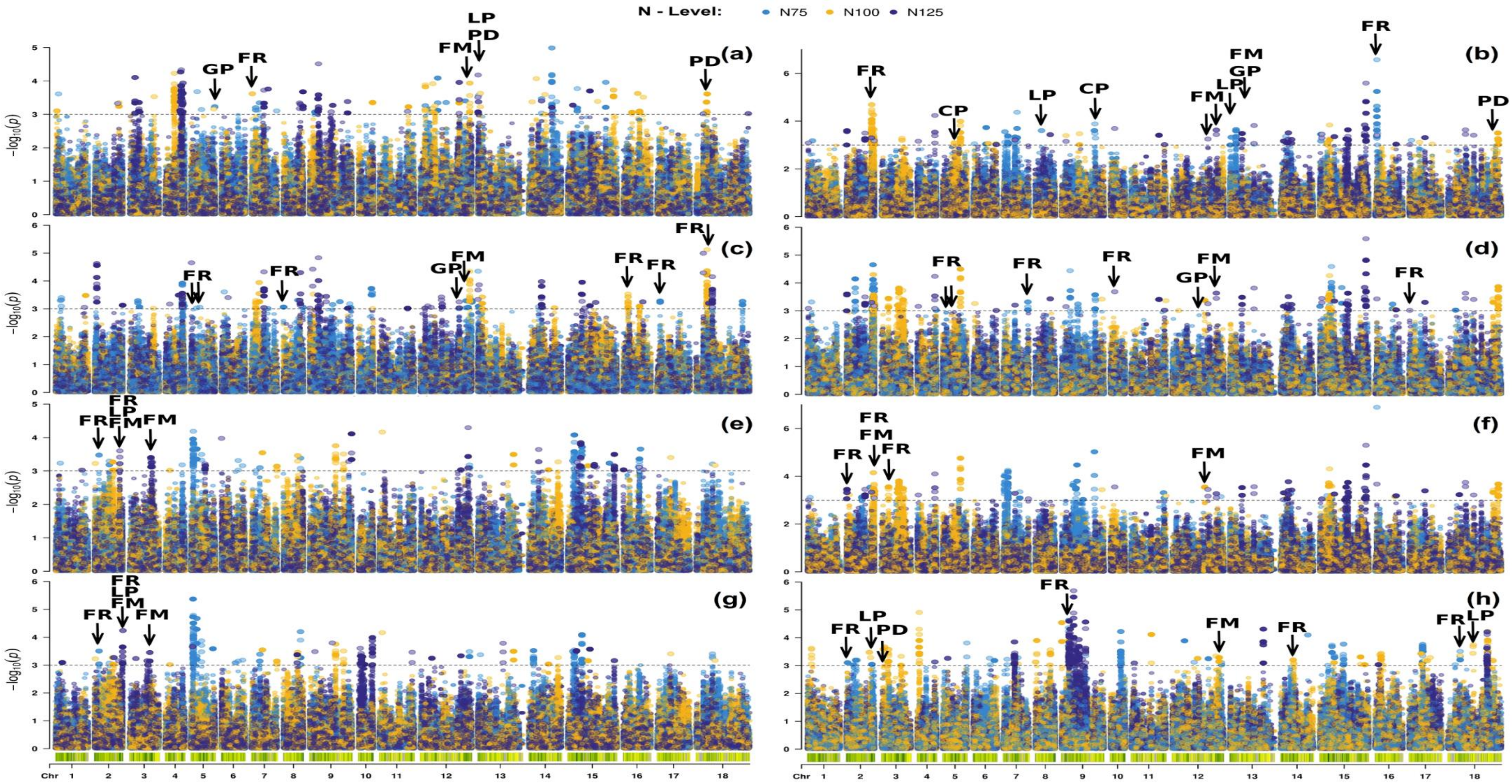

Supplementary Fig. S5. Manhattan plots of flowering traits combined over three levels of N application: a) days to flowering initiation (DFI), b) days to 50% flowering (DFL),c) days to culmination of flowering (DCF),d) maturity (MRT), e) growing degree days (GDD), f) photo-thermal units (PTU) and g) helios thermal unit (HTU), h) plant height (PH). Light blue, yellow and dark blue dot in the Manhattan reflects association at N75, N100 and N125, respectively. Black arrows indicate the predicted genes related various photosynthetic pathways: light perception (LP), circadian pathway (CP), floral meristem identity genes (FM), flowering regulation (FR), GP- gibberellin pathway (GP) and plant development (PD). Dotted black horizontal line are the genome-wide significance threshold [-log10(*p*) > 3.0]. Bottom chromosome colour bar depicts SNP density (yellow colour = low SNPs density and green colour = high SNPs density).
